# 1117. Tazobactam Pharmacokinetic/Pharmacodynamic Target Attainment in Healthy Volunteers and Critically-Ill Hospitalized Patients

**DOI:** 10.1093/ofid/ofab466.1310

**Published:** 2021-12-04

**Authors:** Shamir N Kalaria, Jason M Pogue, Emily Heil, Emily Heil

**Affiliations:** 1 University of Maryland Medical Center, Baltimore, Maryland; 2 College of Pharmacy, University of Michigan, Ann Arbor, Michigan; 3 University of Maryland School of Pharmacy; University of Maryland Medical Center, Baltimore, MD

## Abstract

**Background:**

Pharmacokinetic/pharmacodynamic (PK/PD) targets and attainment are well described for beta-lactams; however, are rarely considered for beta-lactamase inhibitors. Recent evidence suggests that tazobactam (TAZ) target exposures to restore piperacillin bacteriostatic and 1 log 10 bactericidal activity against Enterobacterales are fT> the piperacillin/tazobactam (TZP) MIC of 64% and 77%, respectively. The aim of this study was to evaluate TAZ probability of target attainment (PTA) of a 500 mg every 6-hour dose of tazobactam using population PK data in both healthy volunteers and hospitalized patients.

**Methods:**

PK exposures in 1,000 patients with varying degrees of renal function were simulated using a previously described TAZ PK model developed with data from critically ill infected patients. An identical one-compartment structural model describing TAZ PK using mean population parameters observed in phase 1 PK studies was also used to simulate exposures in healthy volunteers. All simulated patients received 500 mg of TAZ as an intravenous infusion over 30 minutes or as a 3-hour extended-infusion.

**Results:**

The table displays PTA results for patients with an estimated creatinine clearance of 60 mL/min. Based on healthy volunteer data, the highest TZP MIC where ~90% PTA was achieved for bacteriostasis was 1 mg/L and was 0.25 mg/L for bactericidal activity. These were only achieved with extended infusion administration of TAZ. In the cohort of hospitalized patients, >90% PTA of TAZ exposures associated with both bacteriostasis and 1 log kill were achieved up to a MIC of 2 for intermittent infusion and up to 4 mg/L for extended infusion, due to decreased TAZ clearance in hospitalized patients. These values are significantly lower than the CLSI TZP susceptibility breakpoint of 16 mg/L, and PTA rates were lower at increased creatinine clearances.

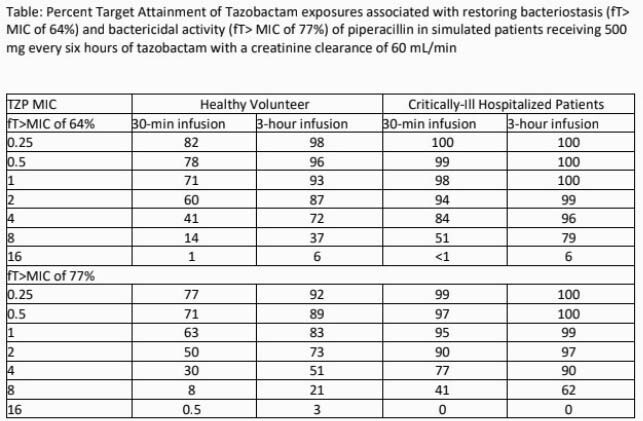

**Conclusion:**

fT>TZP MIC target attainment is poor with maximal package insert tazobactam doses given with piperacillin, even when administered as an extended infusion. These findings have serious implications for the role of TZP in beta-lactamase producing Enterobacterales, including ESBLs, and suggest the current susceptibility breakpoints are 4-32 fold higher than those supported by PK/PD data.

**Disclosures:**

**Jason M Pogue, PharmD, BCPS, BCIDP**, **Merck** (Consultant)**QPex** (Consultant)**Shionogi** (Consultant)**Utility Therapeutics** (Consultant)**VenatoRX** (Consultant) **Emily Heil, PharmD, MS, BCIDP**, Nothing to disclose

